# Acute Pancreatitis as a Sequela of Hypertriglyceridemia Due to Hyperosmolar Hyperglycemic Syndrome

**DOI:** 10.7759/cureus.19640

**Published:** 2021-11-16

**Authors:** Daniel Shor, Samantha Harrison, Keith Anacker, Joan Wiley

**Affiliations:** 1 Family Medicine, Rowan University School of Osteopathic Medicine, Stratford, USA; 2 Internal Medicine, Philadelphia College of Osteopathic Medicine, Philadelphia, USA; 3 Internal Medicine, Rowan University School of Osteopathic Medicine, Stratford, USA; 4 Critical Care Physician, Jefferson Health New Jersey, Cherry Hill, USA

**Keywords:** diabetes, blood glucose, hyperosmolar hypoglycemic syndrome, hypertriglyceridemia, pancreatitis

## Abstract

It is well known that hyperosmolar hyperglycemic state (HHS) can lead to hypertriglyceridemia (HTG), and that HTG can lead to acute pancreatitis. However, few case reports exist of these three conditions occurring simultaneously. In this case report, we describe a 49-year-old female with a past medical history of well-controlled hypertension who presented to the emergency department with abdominal pain and hematemesis after being found minimally responsive at home. Labs and imaging on admission were consistent with acute pancreatitis in the setting of severe HTG. She also had a significantly elevated glucose and serum osmolality consistent with HHS. We suggest the patient had HHS that led to an HTG severe enough to cause acute pancreatitis. These findings may provide insight into HHS as an important predisposing condition to acute pancreatitis.

## Introduction

Acute pancreatitis is an inflammation of the pancreas that can be caused by multiple etiologies. The most common causes of acute pancreatitis include gallstones, alcoholism, certain medications as well as elevated triglyceride levels [1]. Pancreatitis commonly presents as severe abdominal pain that can radiate to the back, with associated nausea and vomiting, and is exacerbated by eating [1]. Diagnosis can usually be made by abdominal imaging which will typically display pancreatic/peripancreatic fluid, edema, and/or necrosis in the setting of elevated amylase and/or lipase [1]. It is not uncommon for hypertriglyceridemia (HTG) to be the cause of acute pancreatitis. However, it is rare for hyperosmolar hyperglycemic state (HHS) to be the cause of an HTG severe enough to precipitate acute pancreatitis. HHS is an acute derangement of metabolism in which a relative deficiency in insulin causes a profound hyperglycemia that results in a state of dehydration without ketoacidosis [2]. It is most commonly associated with type 2 diabetes mellitus and presents with symptoms including thirst, polydipsia, and polyuria [2]. In severe cases, it may cause neurologic deficits, coma, or even death [2]. Management includes aggressive volume repletion as well as intravenous insulin administration [3]. In addition to an HHS, relative insulin deficiency is associated with an elevated serum triglyceride level [2]. This is because insulin inhibits the breakdown of fat within the body’s adipose stores [2]. Therefore, in HHS, there is less insulin available to inhibit the breakdown of fat and more triglycerides are released into the serum.

## Case presentation

A previously healthy 49-year-old female with a past medical history of well-controlled hypertension and body mass index (BMI) of 30.37 kg/m2 presented to the emergency department with altered mental status, abdominal pain, hematemesis, and hypotension. According to family, the patient complained of abdominal pain earlier that morning and was later found at home minimally responsive and recurrently vomiting blood.

**Figure 1:**
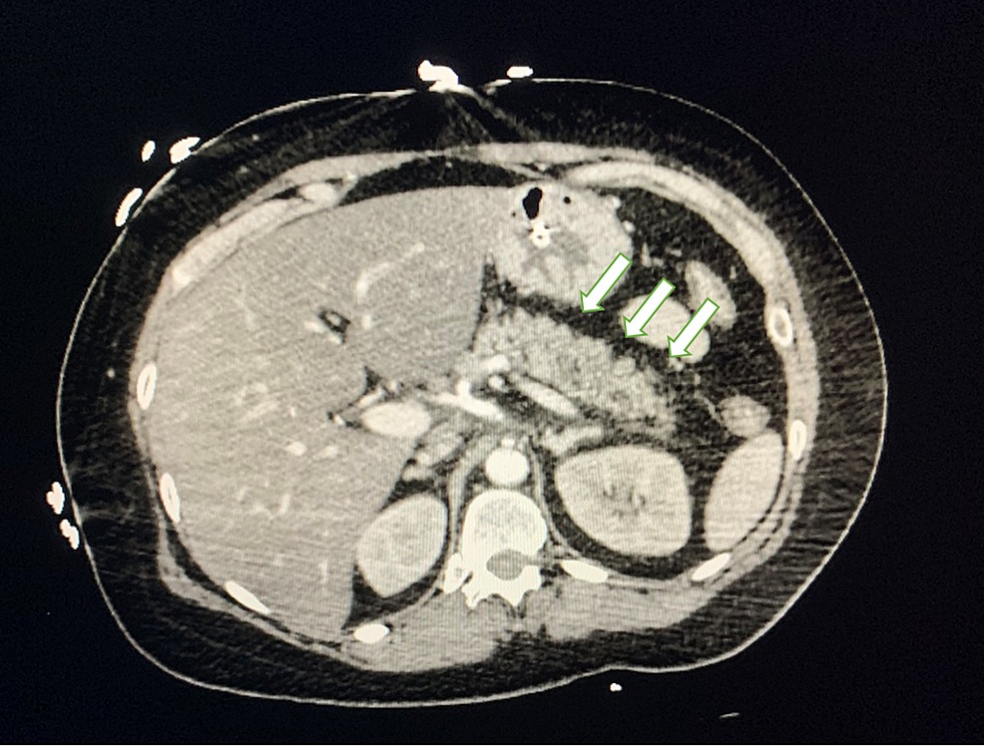
CT chest-abdomen-pelvis with IV contrast Acute pancreatitis with peripancreatic edema and fat stranding (white arrows). No peripancreatic collection or evidence of necrosis.

In the emergency department, the patient’s vitals included a blood pressure of 94/50 mmHg, a temperature of 87.1 Fahrenheit, and a respiratory rate of 34 breaths per minute. The patient was intubated for airway protection. She received 5L of fluid as well as one unit of packed red blood cells for suspected large fluid volume loss. Esophagogastroduodenoscopy was performed and was remarkable for a Mallory-Weiss tear with portohypertensive gastropathy. This was thought to be caused by the repeated vomiting reported by her family. CT scan showed peripancreatic edema and fat stranding, consistent with acute pancreatitis (Figure 1). A repeat CT scan was done to evaluate the progression of her pancreatitis, which showed worsening pancreatitis with developing ascites. Labs were remarkable for a glucose up to 955 mg/dL, hemoglobin A1c (HgbA1c) of 13.7%, and a triglyceride level up to 1608 mg/dL (Table 1). The patient was then placed on an insulin drip for her significantly elevated blood glucose. After her glucose normalized, she was continued on an insulin drip until her triglycerides dropped below 500 mg/dL. The patient was then downgraded to the general medical floor and discharged after being able to tolerate a regular diet without significant pain or discomfort.

**Table 1:** Laboratory values *Specimen markedly hemolyzed, unable to obtain correct results **Initial value was too high to read, thus it was sent to another hospital to get a quantitative value which is the value provided ***Hemoglobin value obtained before transfusion of one unit of packed red blood cells

Laboratory Test	Initial day	2^nd^ day	3^rd^ day	4^th^ day
Lipase (U/L)	728	151	66	
Triglycerides (g/dL)	*	1608**	586	390
Glucose (mg/dL)	955	160	79	
Hemoglobin (g/dL)	14.1***	13.9	13.4	
Hemoglobin A1c (%)	13.7			

## Discussion

There are few reports in the literature of HHS, HTG, and acute pancreatitis occurring concomitantly. Okoli et al. [4] described a case of a 31-year-old previously healthy male patient who presented to the emergency department with complaint of syncope and abdominal pain. He reported six months of fatigue and weakness with an unintentional weight loss of 30 pounds, polyuria, polydipsia, and vision changes, which contrasts from the acute development of symptoms in the patient presented in our case report. Another difference in our case report is that a quantitative triglyceride value was obtained on day two of the patient’s hospital stay, the day after her insulin drip was started. This is because the initial blood sample was hemolyzed, and the next sample was too high to read by the machine in the hospital, so another blood sample had to be sent to a different facility where it was finally read as a quantitative value. Therefore, it is reasonable to believe that the patient had an even higher maximum triglyceride level than the one listed (Table 1).

Giri et al. [5] presents a case report of a 52-year-old patient with obesity and type 2 diabetes who presented with acute pancreatitis after a recent COVID-19 infection. She reportedly had the following labs: blood glucose >1000 mg/dl, creatinine of 1.7, anion gap > 31, elevated beta-hydroxybutyrate, minimal urine ketones, serum bicarbonate of 13, and lipase >2000. However, she did not have elevated triglycerides, which contrasts this case from ours as our patient had severely elevated triglycerides. Giri et al. [5] suggest that pancreatitis in their patient was likely due to a decompensated diabetic states with COVID-19 causing cytokine-mediated damage to the pancreatic beta cells, which led to inflammation of the pancreas and worsening glycemic control, precipitating diabetic ketoacidosis (DKA) or HHS.

Evidence exists in the literature that suggests that insulin-deficient states including HHS and DKA are associated with increased lipolysis and free fatty acid (FFA) release [4]. Higher levels of FFA in the blood lead to increased conversion of FFA to higher density lipoproteins such as very-low-density lipoproteins (VLDLs) which can trigger a state of HTG. HTG is a well-known cause of acute pancreatitis. Studies have shown that HTG with serum TG levels ≥500 mg/dL increases the risk of acute pancreatitis [3]. HTG induced acute pancreatitis is associated with systemic complications and has an estimated mortality rate of 10% and even higher, therefore, early diagnosis and intervention is critical [3]. Although HTG and HHS commonly present together and HTG is a well-established cause of acute pancreatitis, few case reports exist in the literature of the three conditions occurring simultaneously. Because of the lack of literature, this triad may be often missed in the clinical setting. More research is needed to provide insight on the pathophysiology and prevalence of HHS and HTG as a cause of acute pancreatitis.

## Conclusions

HTG is a known cause of acute pancreatitis. In some cases, HHS can result in an HTG severe enough to cause acute pancreatitis. It is important to recognize that the triad of HHS, HTG, and pancreatitis presenting together can represent an overarching syndrome as described in this case report. There are few documented cases in the literature that describe this presentation of HHS and further research needs to be done to make clinicians more aware of it.
